# Modelling phase precession in the hippocampus

**DOI:** 10.1186/1471-2202-16-S1-O18

**Published:** 2015-12-18

**Authors:** Angus Chadwick, Mark van Rossum, Matthew Nolan

**Affiliations:** 1 Institute for Adaptive and Neural Computation, University of Edinburgh, Edinburgh, UK; 2 Centre for Integrative Physiology, University of Edinburgh, Edinburgh, UK

## 

The activity of cells in the rodent hippocampus is strongly modulated by both the location of the animal and the ongoing theta oscillation. Place cells, but not interneurons, show a strong spatial modulation of their firing rates, while both place cells and interneurons exhibit phase precession, a phenomenon whereby they spike at a faster frequency than the LFP theta oscillation, causing their spikes to shift to an earlier phase of this rhythm on each successive cycle [1-3]. Despite extensive research into this phenomenon, the mechanisms underlying phase precession remain unclear.

Place cells and interneurons are reciprocally connected in the CA1 region of the hippocampus. The interneurons receive pacemaker input from the medial septum, which entrains theta oscillations in the circuit. We tested whether a minimal model based on this architecture could produce phase precession in place cells and interneurons. Specifically, we simulated a single place cell and interneuron, which interact synaptically. The interneuron was driven with a constant depolarising input which generates tonic spiking, as well as a weak theta oscillation which entrains this spiking activity. The place cell received a depolarising input which is only active at a certain location in the environment, representing the place field.

We found that phase precession in both the place cell and interneuron emerges naturally in this model. When the animal is outside of the place field, the interneuron is fully entrained to the pacemaker theta oscillation, and the place cell is rhythmically inhibited, resulting in subthreshold theta oscillations. When the animal enters the place field, the place cell begins to spike, which perturbs the interneuron and causes it to transiently fire at a frequency higher than the pacemaker input. In turn, the spiking of the interneuron entrains the place cell, generating phase precession in the coupled pair.

Generalisation of this model to the network level reveals important constraints. In particular, as there are far fewer interneurons than place cells in CA1, it is necessary that the same interneuron is coupled to multiple place cells. In our model, a single interneuron can successfully couple to multiple place cells to generate phase precession, provided that their place fields do not overlap. This poses constraints on the possible place field mappings in such a network, and places limits on the fraction of place cells which can be active in a single environment. When working within these limits, the network can flexibly generate phase precession, both in linear and open environments, across a vast number of distinct place field mappings.

Our model has several advantages over existing models. First, our model generates phase precession through the intrinsic dynamics of the circuit, without the need for velocity controlled oscillators upstream. Second, our model can generate omnidirectional phase precession in open environments, without additional inputs from head direction cells. Finally, our model generates phase precession independently in each cell, and therefore allows spatial representations to be flexibly remapped without detriment to the temporal coding of spatial trajectories in the population [4].
